# Body weight variability and the risk of cardiovascular outcomes in patients with nonalcoholic fatty liver disease

**DOI:** 10.1038/s41598-021-88733-3

**Published:** 2021-04-28

**Authors:** Mi Na Kim, Kyungdo Han, Juhwan Yoo, Yeonjung Ha, Young Eun Chon, Ju Ho Lee, Tracey G. Simon, Andrew T. Chan, Seong Gyu Hwang

**Affiliations:** 1 Division of Gastroenterology, Department of Internal Medicine, CHA Bundang Medical Center, CHA University School of Medicine, 59 Yatap-ro, Bundang-gu, Seongnam, 13496 Republic of Korea; 2 Clinical and Translational Hepatology Laboratory, Seongnam, Republic of Korea; 3 Department of Statistics and Actuarial Science, Soongsil University, Seoul, Republic of Korea; 4 Department of Biomedicine and Health Science, The Catholic University of Korea, Seoul, Republic of Korea; 5 Clinical and Translational Epidemiology Unit, Massachusetts General Hospital and Harvard Medical School, Boston, MA USA; 6 Division of Gastroenterology, Massachusetts General Hospital and Harvard Medical School, Boston, MA USA; 7 Liver Center, Division of Gastroenterology, Department of Medicine, Massachusetts General Hospital and Harvard Medical School, Boston, MA USA

**Keywords:** Cardiology, Gastroenterology

## Abstract

We investigated the association between body weight variability and the risks of cardiovascular disease and mortality in patients with nonalcoholic fatty liver disease (NAFLD) using large-scale, nationwide cohort data. We included 726,736 individuals with NAFLD who underwent a health examination between 2009 and 2010. NAFLD was defined as a fatty liver index ≥ 60, after excluding significant alcohol intake, viral hepatitis, and liver cirrhosis. Body weight variability was assessed using four indices, including variability independent of the mean (VIM). During a median 8.1-year follow-up, we documented 11,358, 14,714, and 22,164 cases of myocardial infarction (MI), stroke, and all-cause mortality, respectively. Body weight variability was associated with an increased risk of MI, stroke, and mortality after adjusting for confounding variables. The hazard ratios (HRs) (95% confidence intervals) for the highest quartile, compared with the lowest quartile, of VIM for body weight were 1.15 (1.10–1.20), 1.22 (1.18–1.26), and 1.56 (1.53–1.62) for MI, stroke, and all-cause mortality, respectively. Body weight variability was associated with increased risks of MI, stroke, and all-cause mortality in NAFLD patients. Appropriate interventions to maintain a stable weight could positively affect health outcomes in NAFLD patients.

## Introduction

Nonalcoholic fatty liver disease (NAFLD) is the most prevalent liver disease, with an estimated overall prevalence of 25%^1^, and its prevalence is increasing worldwide^2^. NAFLD encompasses a clinicopathological spectrum ranging from simple steatosis to nonalcoholic steatohepatitis^2,3^. Nonalcoholic steatohepatitis is the more aggressive form of NAFLD, which is characterized by steatosis, hepatocyte injury and inflammation, with or without fibrosis. It can progress to cirrhosis and the associated complications^4^. NAFLD is related to the development of hepatocellular carcinoma or liver failure^5^ and is also associated with the risk of developing extra-hepatic manifestations, such as cardiovascular disease (CVD), chronic kidney disease, and certain extra-hepatic malignancies^6^. Among these, CVD is the leading cause of increased long-term morbidity and mortality in NAFLD patients^7^.

The primary treatment of NAFLD is lifestyle changes through diet and exercise modifications to promote significant weight loss^4,8–10^. A weight loss of 7–10% reduces liver fat content, liver inflammation, and fibrosis in overweight and obese patients with nonalcoholic steatohepatitis^11^. Although lean NAFLD subjects are of normal weight, weight loss with lifestyle changes induces the resolution of NAFLD and improvement in steatosis^10^. However, weight loss is rarely sustainable, and a substantial proportion of NAFLD patients who try weight loss experience weight regain^12^. A pooled follow-up analysis of three large weight-loss trials showed that only 23% maintained weight loss during the third year^13^. Weight regain after weight loss results from homeostatic feedback mechanisms, including change in hunger and satiety hormones and altered characteristics of adipocytes to store more energy during periods of weight loss^14^.

Body weight variability, which is also termed weight fluctuation or weight cycling, is defined as repeated weight loss and subsequent regain. In several epidemiologic studies, body weight variations have been associated with increased risks of future cardiovascular events and mortality^15–17^. Given that the vast majority of NAFLD patients have difficulty maintaining weight loss, and because CVD is closely associated with NAFLD, investigating of the influence of body weight variability on CVD and mortality in NAFLD patients is crucial to prevent the deleterious consequences of NAFLD.

Therefore, we investigated the association between body weight variability and the risks of CVD and mortality in patients with NAFLD using large-scale, nationwide cohort data.

## Methods

### Study population

We used a representative sample cohort provided by the Korean National Health Insurance Service (NHIS) of the National Health Insurance Corporation (NHIC). Approximately 97% of the South Korean population is insured by the NHIS (the sole insuring organization). Standardized health examinations are recommended for enrollees in the NHIS. The NHIC releases data containing various types of individual health information^18^.

From this cohort, we enrolled 17,539,992 individuals who underwent health examinations between January 2009 and December 2010. Subjects who met the following criteria were excluded based on our protocol: (1) received a health examination fewer than three times within 5 years of enrollment (n = 9,163,132), (2) aged < 20 years (n = 106), (3) any missing data (n = 319,788), (4) fatty liver index (FLI) < 60 (n = 7,008,442), (4) significant alcohol consumption (defined as alcohol intake ≥ 30 g/day for men or ≥ 20 g/day for women) (n = 198,203), (5) prior hepatocellular carcinoma history (n = 576), (6) viral hepatitis or liver cirrhosis (n = 113,295) or (7) prior diagnosis of myocardial infarction (MI) or stroke (n = 9714). The remaining 726,736 participants were included in the final analysis (Supplementary Fig. 1) and were followed up until death or December 31, 2017. This study protocol was reviewed and approved by the Institutional Review Board of CHA University (IRB no. 2020-07-073). The NHIS database was constructed with anonymized data following strict confidentiality guidelines, so the requirement for written informed consent was waived by the Institutional Review Board of CHA University. We confirm that all methods were performed in accordance with the principles expressed in the Declaration of Helsinki.

### Definitions of NAFLD and liver fibrosis

NAFLD was defined using the FLI, which is a previously validated predictive marker of fatty liver^19^. A FLI ≥ 60 is indicative of NAFLD. The fibrotic burden of subjects with NAFLD was assessed using the BARD score, a previously validated predictive marker of liver fibrosis. Advanced liver fibrosis (fibrosis ≥ stage 3) is defined as a BARD score ≥ 2^20^. Supplementary Table 1 summarizes these prediction models.

### Anthropometric measurements and indices of body weight variability

Body weight (kg), height (m), and waist circumference (cm) were measured at each visit. Body mass index (BMI) was calculated as body weight divided by height squared. Obesity was defined as a BMI ≥ 25 kg/m^2^ based on the World Health Organization recommendation for Asian populations^21^. Our analysis used a minimum of three body weight measurements taken within 5 years before the index date (including the examination on the index date). Body weight variability was determined using the following four indices: (1) variability independent of the mean (VIM), (2) standard deviation (SD), (3) coefficient of variation (CV), and (4) average real variability (ARV). VIM was calculated as 100 × SD/mean^β^, where β is the regression coefficient, based on the ln of the SD over the ln of the mean^22^. ARV is based on the average absolute difference between consecutive values. The following formula was used to calculate ARV in this study:

$$ {\text{ARV}} = \frac{1}{{N - 1}}\sum\limits_{{k = 1}}^{{N - 1}} {\left| {Value_{{k + 1}}  - Value_{k} } \right|} , $$

where *n* denotes the number of anthropometric measurements^17^.

### Study outcomes and follow-up

The primary endpoints of this study were incident MI, stroke, and all-cause mortality. Using our claims database, MI was determined as ICD-10-CM code I21 or I22 during hospitalization, or these codes were recorded at least twice. Stroke was defined as ICD-10-CM code I63 or I64 during hospitalization according to brain magnetic resonance imaging or brain computed tomography. Mortality data were obtained from the Korean National Statistical Office. Follow-up was completed at the occurrence of cardiovascular events (MI or stroke) or all-cause death.

### Definition of covariates

Demographic and lifestyle data were obtained using a self-reported questionnaire. Smoking status was classified as nonsmoker, former smoker, or current smoker. Regular exercise was defined as strenuous physical activity for ≥ 20 min at least three times per week or moderate physical activity for ≥ 30 min at least five times per week. Income level was dichotomized into < 25% or ≥ 25%. Data from health examinations, such as blood pressure (BP) and laboratory measurements, were provided. Diabetes mellitus (DM) was defined as a fasting plasma glucose level ≥ 126 mg/dL or having at least one prescription claim per year for an antidiabetic medication under the ICD-10 codes E11-E14. Systolic BP ≥ 140 mmHg, diastolic BP ≥ 90 mmHg, or at least one prescription claim per year for antihypertensive medication under ICD-10-CM codes I10-I13 and I15 was defined as having hypertension. Dyslipidemia was defined as a serum total cholesterol level ≥ 240 mg/dL^23^ or at least one prescription claim per year for a lipid-lowering medication under ICD-10-CM code E78. Chronic kidney disease was defined as an estimated glomerular filtration rate < 60 mL/min/1.73 m^2^^24^.

### Statistical analysis

The baseline characteristics of the study participants according to the VIM categories of body weight are presented as means ± SD for continuous variables and numbers (percentages) for categorical variables. Analysis of variance was used to compare continuous variables, and the chi-square test was used to compare categorical variables. The incidence rate was calculated by dividing the number of events by 1000 person-years. The association between body weight variability and the risk of the study outcome was evaluated using body weight variability as both a categorical and continuous variable. When body weight variability was taken as a categorical variable, subjects were divided into quartiles, and outcomes were evaluated for all quartiles. The association between the body weight variability quartile and the risk of the study outcome was analyzed using Cox proportional hazards regression. Additionally, we analyzed the association between the body weight variability as a continuous variable and the risk of the study outcome. Hazard ratios (HRs) and 95% confidence intervals (CIs) were calculated using the lowest quartile as the reference. In the multivariate-adjusted models, model 1 was adjusted for age and sex, model 2 was adjusted for age, sex, smoking status, alcohol consumption, physical activity, DM, hypertension, dyslipidemia, and chronic kidney disease; and model 3 was further adjusted for baseline BMI on the index date in addition to the variables adjusted in model 2. Subgroup analyses according to age, sex, smoking status, DM, hypertension, dyslipidemia, alcohol consumption, physical activity, and baseline BMI were performed. *P* values for interaction were calculated using Cox regression analyses. All statistical analyses were performed using SAS version 9.3 software (SAS Institute, Cary, NC, USA).

## Results

### Baseline characteristics

Table 1 demonstrates the baseline characteristics of the study population (n = 726,736) according to the quartiles of VIM for body weight. The mean waist circumference and BMI was highest in quartile 4, and lower in the lower quartiles of VIM. The mean age, the rates of DM, hypertension, and chronic kidney disease, and the proportion of those who exercised regularly were highest in quartile 1 and decreased with increasing quartile of VIM for body weight. The mean values on liver function tests, such as aspartate aminotransferase, alanine aminotransferase, and gamma-glutamyl transpeptidase levels, were lower in the higher quartiles of VIM for body weight. The mean total cholesterol and high-density lipoprotein-cholesterol levels increased from the lowest to highest quartile. The mean values of FLI were higher in the higher quartiles. The proportion of patients with significant liver fibrosis was lower in the higher quartiles.

**Table 1:** Baseline characteristics of study population according to VIM for body weight category.

	VIM				*P* value
	Q1 (n = 181,907)	Q2 (n = 181,484)	Q3 (n = 181,668)	Q4 (n = 181,677)	
**Variables**					
Demographic variables					
Age (years)	49.74 ± 11.72	48.31 ± 11.82	47.17 ± 12.29	44.88 ± 13.63	< 0.001
Male sex	156,566 (86.07)	157,615 (86.85)	156,428 (86.11)	147,713 (81.31)	< 0.001
Height (cm)	167.88 ± 8.08	168.21 ± 8.24	168.44 ± 8.32	168.33 ± 8.95	< 0.001
Weight (kg)	77.65 ± 9.39	78.22 ± 10.14	79.01 ± 10.37	80.77 ± 11.67	< 0.001
Waist circumference (cm)	91.67 ± 6.15	91.67 ± 6.34	91.95 ± 6.51	92.93 ± 7.12	< 0.001
BMI (kg/m^2^)	27.53 ± 2.57	27.61 ± 2.67	27.81 ± 2.79	28.47 ± 3.23	< 0.001
SD of weight	0.82 ± 0.32	1.57 ± 0.26	2.35 ± 0.37	4.44 ± 2.07	< 0.001
CV of weight	1.06 ± 0.4	2.03 ± 0.25	3.02 ± 0.35	5.72 ± 2.68	< 0.00
VIM of weight	0.7 ± 0.26	1.34 ± 0.17	2 ± 0.23	3.79 ± 1.76	< 0.001
ARV of weight	0.96 ± 0.49	1.83 ± 0.58	2.63 ± 0.82	4.72 ± 2.65	< 0.001
Systolic BP (mm Hg)	129.31 ± 13.89	129.33 ± 13.82	129.31 ± 13.89	129.41 ± 14.04	0.107
Diastolic BP (mm Hg)	81.17 ± 9.56	81.34 ± 9.57	81.29 ± 9.59	81.24 ± 9.66	< 0.001
Hypertension	81,302 (44.69)	78,290 (43.14)	76,016 (41.84)	72,632 (39.98)	< 0.001
DM	31,953 (17.57)	30,375 (16.74)	29,719 (16.36)	28,465 (15.67)	< 0.001
Dyslipidemia	50,961 (28.01)	51,412 (28.33)	50,516 (27.81)	47,841 (26.33)	< 0.001
Chronic kidney disease	11,499 (6.32)	10,866 (5.99)	10,545 (5.8)	10,471 (5.76)	< 0.001
Smoking status					
Current	71,104 (39.09)	74,639 (41.13)	75,353 (41.48)	73,085 (40.23)	< 0.001
Former	42,350 (23.28)	41,500 (22.87)	41,097 (22.62)	38,241 (21.05)	< 0.001
Never	68,453 (37.63)	65,345 (36.01)	65,218 (35.9)	70,351 (38.72)	< 0.001
Alcohol intake (g/day)					
None	64,203 (35.29)	62,346 (34.35)	63,444 (34.92)	69,785 (38.41)	< 0.001
Mild	117,704 (64.71)	119,138 (65.65)	118,224 (65.08)	111,892 (61.59)	< 0.001
Central obesity^a^	119,511 (65.7)	118,280 (65.17)	120,807 (66.5)	128,985 (71)	< 0.001
Obesity^b^	155,338 (85.39)	154,046 (84.88)	156,501 (86.15)	159,887 (88.01)	< 0.001
Regular exerciser	34,264 (18.84)	33,677 (18.56)	32,592 (17.94)	29,873 (16.44)	< 0.001
Low income	32,217 (17.71)	32,043 (17.66)	32,825 (18.07)	35,571 (19.58)	< 0.001
Laboratory variables					
Fasting blood glucose (mg/dL)	106.04 ± 27.64	105.69 ± 28.5	105.43 ± 29.26	104.91 ± 31.08	< 0.001
Total cholesterol (mg/dL)	211.56 ± 37.48	212.09 ± 37.6	212.43 ± 37.92	212.66 ± 38.44	< 0.001
Triglyceride (mg/dL)	227.7 (227.23–228.18)	229.89 (229.41–230.38)	227.37 (226.89–227.86	218.7 (218.22–219.18)	< 0.00
HDL cholesterol (mg/dL)	47.72 ± 14.71	47.73 ± 14.42	47.93 ± 14.78	48.41 ± 14.76	< 0.001
LDL cholesterol (mg/dL)	114.49 ± 37.63	114.31 ± 37.82	114.93 ± 37.98	116.29 ± 38.03	< 0.00
Serum creatinine (mg/dL)	1.08 ± 0.79	1.09 ± 0.81	1.08 ± 0.8	1.06 ± 0.77	< 0.001
eGFR (mL/min/1.73 m^2^)	85.43 ± 43.87	85.7 ± 42.27	86.47 ± 44.66	88.03 ± 45.64	< 0.001
Aspartate aminotransferase (IU/L)	30.53 (30.48–30.58)	30.92 (30.87–30.97)	31.31 (31.26–31.37)	31.88 (31.82–31.94)	< 0.00
Alanine aminotransferase (IU/L)	36.96 (36.88–37.05)	37.9 (37.81–37.99)	38.9 (38.81–38.99)	40.3 (40.2–40.41)	< 0.001
Gamma-glutamyl transpeptidase (IU/L)	64.22 (64.03–64.41)	65.54 (65.34–65.73)	65.1 (64.91–65.3)	62.38 (62.18–62.57)	< 0.001
Fatty liver index	73.2 ± 9.27	73.7 ± 9.51	74.09 ± 9.7	75.08 ± 10.13	< 0.001
Liver fibrosis					
BARD score					
0	42,877 (23.57)	42,351 (23.34)	42,020 (23.13)	37,043 (20.39)	< 0.001
1	36,817 (20.24)	40,256 (22.18)	43,801 (24.11)	52,115 (28.69)	< 0.001
2	55,303 (30.4)	53,050 (29.23)	49,291 (27.13)	41,993 (23.11)	< 0.001
3	39,742 (21.85)	39,136 (21.56)	39,579 (21.79)	42,911 (23.62)	< 0.001
4	7168 (3.94)	6691 (3.69)	6977 (3.84)	7615 (4.19)	< 0.001
Significant liver fibrosis (defined by BARD score ≥ 2)	102,213 (56.19)	98,877 (54.48)	95,847 (52.76)	92,519 (50.92)	< 0.001

Data are presented as mean ± SD or number (percentage).

^a^Central obesity was defined as waist circumference ≥ 90 cm in men and ≥ 85 cm in women.

^b^Obesity was defined as BMI ≥ 25 kg/m^2^.

### Association between body weight variability and the risks of outcomes

During a median 8.1-year follow-up, we documented 11,358, 14,714, and 22,164 cases of MI, stroke, and all-cause mortality, respectively. Table 2 shows the risks of MI, stroke, and all-cause mortality according to the quartile of VIM for body weight. After adjusting for age and sex (model 1), the HRs for MI, stroke, and all-cause mortality were significantly greater in the higher quartiles of VIM for body weight (all *P* for trend < 0.001). These significant and positive associations remained after adjusting for the covariates in model 2, and further adjusting for baseline BMI (*P* for trend = 0.0001 for MI and < 0.001 for stroke and mortality). After further adjusting for baseline BMI in model 3, the HRs (95% CI) were 1.09 (1.03–1.14) for MI, 1.22 (1.17–1.28) for stroke, and 1.53 (1.47–1.58) for all-cause mortality in quartile 4, compared with quartile 1. The risks for MI, stroke, and all-cause mortality were significantly higher in the higher quartiles of other parameters of body weight variability (SD, CV, and ARV) (all *P* for trend < 0.001). (Supplementary Table 2) In addition, when we analyzed the association between the body weight variability as a continuous variable and the risks for MI, stroke, and all-cause mortality, all the parameters of body weight variability demonstrated the significant positive association of increased risks for MI, stroke, and all-cause mortality (all *P* < 0.05) (Supplementary Table 3).

**Table 2:** Risks of outcomes with respect to quartiles of VIM for body weight.

	n	Event	Person-years	Incidence-rate^a^	HR (95% CI)		
					Model 1^b^	Model 2^c^	Model 3^d^
**Myocardial infarction**							
Q1	181,907	2959	1,426,357.7	2.07451	1 (Ref.)	1 (Ref.)	1 (Ref.)
Q2	181,484	2830	1,435,701.79	1.97116	1.02 (0.97, 1.08)	1.01 (0.96, 1.07)	1.01 (0.96, 1.07)
Q3	181,668	2901	1,432,967.52	2.02447	1.11 (1.05, 1.16)	1.09 (1.04, 1.15)	1.09 (1.04, 1.15)
Q4	181,677	2668	1,423,773.15	1.87389	1.11 (1.06, 1.17)	1.09 (1.03, 1.14)	1.09 (1.03, 1.14)
*P* for trend					< 0.0001	0.0001	0.0002
**Stroke**							
Q1	181,907	3811	1,423,641.67	2.67694	1 (Ref.)	1 (Ref.)	1 (Ref.)
Q2	181,484	3544	1,433,205.76	2.47278	1.02 (0.98, 1.07)	1.02 (0.97, 1.06)	1.01 (0.97, 1.06)
Q3	181,668	3577	1,430,396.39	2.50071	1.09 (1.05, 1.14)	1.08 (1.03, 1.13)	1.08 (1.03, 1.13)
Q4	181,677	3782	1,420,030.07	2.66332	1.24 (1.19, 1.30)	1.22 (1.17, 1.28)	1.22 (1.17, 1.28)
*P* for trend					< 0.0001	< 0.0001	< 0.0001
**All-cause mortality**							
Q1	181,907	5242	1,436,156.4	3.65002	1 (Ref.)	1 (Ref.)	1 (Ref.)
Q2	181,484	5091	1,444,803.25	3.52366	1.08 (1.04, 1.13)	1.08 (1.03, 1.12)	1.07 (1.03, 1.11)
Q3	181,668	5461	1,442,152.91	3.7867	1.23 (1.19, 1.28)	1.22 (1.17, 1.26)	1.22 (1.17, 1.26)
Q4	181,677	6370	1,432,023.1	4.44825	1.55 (1.49, 1.61)	1.52 (1.47, 1.58)	1.53 (1.47, 1.58)
*P* for trend					< 0.0001	< 0.0001	< 0.0001

^a^Incidence per 1000 person-years.

^b^Model 1 was adjusted for age and sex.

^c^Model 2 was further adjusted for age, sex, smoking status, alcohol consumption, physical activity, hypertension, diabetes, dyslipidemia, chronic kidney disease.

^d^Model 3 was further adjusted for age, sex, smoking status, alcohol consumption, physical activity, hypertension, diabetes, dyslipidemia, chronic kidney disease, and baseline BMI.

### Association between body weight variability and the risk outcomes according to advanced liver fibrosis

The risks of MI, stroke, and all-cause mortality according to the quartile of VIM for body weight were separately analyzed according to advanced liver fibrosis. (Table 3) After adjusting for covariates and baseline BMI (model 3), the risk for MI was significantly higher in the higher quartiles of VIM for body weight only in the group with advanced fibrosis (*P* for trend = 0.0002). The risks for stroke and all-cause mortality were significantly higher in the higher quartiles of VIM for body weight, regardless of advanced fibrosis (all *P* for trend < 0.05).

**Table 3:** Risks of outcomes with respect to quartiles of VIM for body weight according to the presence of significant liver fibrosis.

Significant fibrosis (defined by BARD score ≥ 2)		HR (95% CI)						
		n	Event	Person-years	Incidence-rate^a^	Model 1^b^	Model 2^c^	Model 3^d^
Yes	**Myocardial infarction**							
	Q1	102,213	1951	797,689.77	2.44581	1 (Ref.)	1 (Ref.)	1 (Ref.)
	Q2	98,877	1837	778,255.33	2.36041	1.024 (0.96, 1.091)	1.014 (0.952, 1.081)	1.014 (0.951, 1.081)
	Q3	95,847	1873	750,862.74	2.49446	1.112 (1.044, 1.185)	1.093 (1.026, 1.165)	1.093 (1.026, 1.165)
	Q4	92,519	1787	718,596.99	2.48679	1.145 (1.074, 1.221)	1.111 (1.042, 1.185)	1.111 (1.042, 1.185)
*P* for trend						< 0.0001	0.0002	0.0002
No	Q1	79,694	1008	628,667.93	1.60339	1 (Ref.)	1 (Ref.)	1 (Ref.)
	Q2	82,607	993	657,446.47	1.51039	1.02 (0.935, 1.114)	1.012 (0.927, 1.105)	1.012 (0.927, 1.105)
	Q3	85,821	1028	682,104.79	1.5071	1.092 (1, 1.191)	1.081 (0.991, 1.179)	1.079 (0.989, 1.178)
	Q4	89,158	881	705,176.15	1.24933	1.052 (0.96, 1.152)	1.037 (0.947, 1.136)	1.034 (0.943, 1.133)
*P* for trend						0.1174	0.2086	0.2361
Yes	**Stroke**							
	Q1	102,213	2797	794,988.79	3.51829	1 (Ref.)	1 (Ref.)	1 (Ref.)
	Q2	98,877	2532	775,941.88	3.26313	1.001 (0.949, 1.056)	0.993 (0.941, 1.048)	0.991 (0.939, 1.045)
	Q3	95,847	2622	748,131.78	3.50473	1.104 (1.046, 1.164)	1.087 (1.031, 1.147)	1.087 (1.03, 1.146)
	Q4	92,519	2919	714,856.81	4.08334	1.295 (1.229, 1.364)	1.265 (1.2, 1.332)	1.269 (1.204, 1.336)
*P* for trend						< 0.0001	< 0.0001	< 0.0001
No	Q1	79,694	1014	628,652.89	1.61297	1 (Ref.)	1 (Ref.)	1 (Ref.)
	Q2	82,607	1012	657,263.88	1.53972	1.082 (0.992, 1.18)	1.079 (0.988, 1.177)	1.079 (0.988, 1.177)
	Q3	85,821	955	682,264.61	1.39975	1.073 (0.982, 1.172)	1.068 (0.977, 1.166)	1.068 (0.977, 1.167)
	Q4	89,158	863	705,173.26	1.22381	1.11 (1.013, 1.216)	1.103 (1.007, 1.208)	1.103 (1.007, 1.208)
*P* for trend						0.0347	0.0492	0.0484
Yes	**All-cause mortality**							
	Q1	102,213	3978	804,159.05	4.94678	1 (Ref.)	1 (Ref.)	1 (Ref.)
	Q2	98,877	3831	784,097.43	4.88587	1.08 (1.033, 1.129)	1.07 (1.024, 1.119)	1.063 (1.017, 1.111)
	Q3	95,847	4148	756,699.64	5.4817	1.244 (1.191, 1.299)	1.222 (1.17, 1.276)	1.218 (1.166, 1.272)
	Q4	92,519	4993	724,054.16	6.89589	1.582 (1.517, 1.649)	1.541 (1.477, 1.606)	1.541 (1.478, 1.607)
*P* for trend						< 0.0001	< 0.0001	< 0.0001
No	Q1	79,694	1264	631,997.35	2.00001	1 (Ref.)	1 (Ref.)	1 (Ref.)
	Q2	82,607	1260	660,705.82	1.90705	1.088 (1.006, 1.176)	1.085 (1.004, 1.173)	1.085 (1.003, 1.173)
	Q3	85,821	1313	685,453.27	1.91552	1.196 (1.107, 1.292)	1.191 (1.102, 1.287)	1.193 (1.104, 1.289)
	Q4	89,158	1377	707,968.94	1.945	1.45 (1.343, 1.565)	1.444 (1.337, 1.558)	1.449 (1.342, 1.564)
*P* for trend						< 0.0001	< 0.0001	< 0.0001

^a^Incidence per 1000 person-years.

^b^Model 1 was adjusted for age and sex.

^c^Model 2 was further adjusted for age, sex, smoking status, alcohol consumption, physical activity, hypertension, diabetes, dyslipidemia, chronic kidney disease.

^d^Model 3 was further adjusted for age, sex, smoking status, alcohol consumption, physical activity, hypertension, diabetes, dyslipidemia, chronic kidney disease, and baseline BMI.

**Figure 1:**
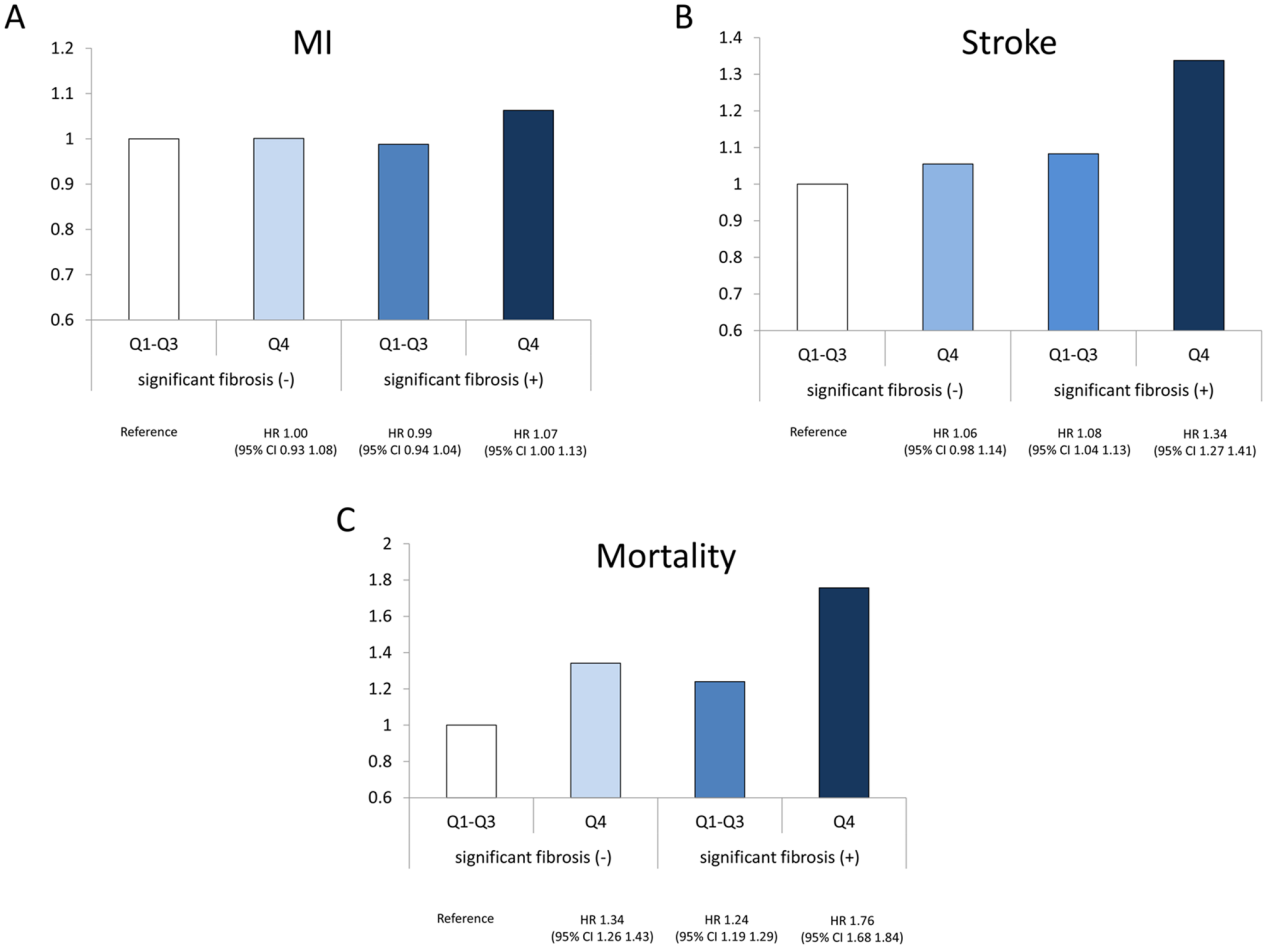
Risks of MI (**A**), stroke (**B**), and all-cause mortality (**C**) based on the existence of significant liver fibrosis or the highest quartile level (Q4) of VIM for body weight.

Figure 1 presents the risks for MI, stroke, and all-cause mortality according to the joint analysis of advanced liver fibrosis and the quartile 4 of VIM. Compared with individuals who did not have either advanced liver fibrosis or quartile 4 of VIM for body weight, those with both advanced liver fibrosis and quartile 4 of VIM for body weight had the highest HRs for all outcomes (for MI: HR 1.07; 95% CI 1.00–1.13; for stroke: HR 1.45; 95% CI 1.27–1.41; and for all-cause mortality: 1.76; 95% CI 1.68–1.84).

### Subgroup analyses

Supplementary Table 3 shows the results of subgroup analyses comparing the risks for the outcomes between quartiles 4 and 1–3 of VIM for body weight. The associations of VIM for body weight with MI and all-cause mortality revealed significant interactions with age (*P* for interaction = 0.0007 and 0.037, respectively). The association between VIM for body weight and stroke was stronger in nonsmokers than in former or current smokers (*P* for interaction = 0.0095). The associations between VIM for body weight and the risks of stroke and all-cause mortality were more prominent in non-obese individuals than in obese individuals (*P* for interaction = 0.0283 and 0.0184, respectively) (Supplementary Table 3).

### Sensitivity analyses

Our findings were robust across the sensitivity analyses. The findings were similar after excluding the study outcomes diagnosed within 3 years of follow-up (Supplementary Table 4). We also repeated the analyses after excluding subjects with cancer, and this did not affect the main results (Supplementary Table 5).

## Discussion

This study investigated the associations between body weight variability and the risks of MI, stroke, and all-cause mortality in NAFLD patients. The associations were independent of traditional CVD and mortality risk factors. The risks of CVD and all-cause mortality among subjects with NAFLD were highest in those with both advanced liver fibrosis and the greatest body weight variability.

Weight loss reduces intrahepatic fat content and improves liver enzyme levels^11,25^. Furthermore, greater weight loss is associated with greater improvements in histological steatosis, hepatocyte ballooning, and lobular inflammation^11,25^. Based on these findings, a 7–10% weight loss is the recommended target when managing overweight or obese NAFLD patients^4,12^. Nevertheless, weight loss achieved intentionally tends to be transient, with subsequent weight gain, even in those motivated enough to participate in a long-term clinical trial^26,27^. Such weight regain attenuates the improvements in fibrosis in patients with NAFLD^28^. However, the long-term health outcomes of weight variability in patients with NAFLD have not been investigated.

High body weight variability has been associated with increased risks of cardiovascular events and mortality in the general population. A recent meta-analysis showed that weight fluctuations are associated with increased risks of CVD (relative risk, 1.49; 95% CI 1.26–1.76; *P* < 0.001) and mortality (relative risk, 1.41; 95% CI 1.27–1.57; *P* < 0.001)^15^. Another meta-analysis demonstrated that the pooled overall HR for all-cause mortality in the group with the greatest weight fluctuations compared with the least was 1.45 (95% CI 1.29–1.63)^29^. A similar trend was reported in studies conducted in patients with underlying disease, such as coronary artery disease^16^, DM^17,30^ and cancer^31,32^. In a post hoc analysis of a randomized controlled trial, patients with coronary artery disease in the greatest quintile of body weight variability had 85% and 124% greater risks of cardiovascular events and mortality, respectively^16^. However, there is little evidence of a similar association between weight variability and long-term health outcomes in NAFLD patients. Our data involved more than 720,000 patients with NAFLD and showed that body weight variability was associated with a significant increase in the risks of cardiovascular events and death. Greater body weight variability was associated with higher CVD and mortality rates.

It is hypothesized that weight regain after weight loss is due to decreased total daily energy expenditure and increased hunger accompanied by a weight-reduced state called metabolic adaptation^33^. Metabolic adaptation manifest as enhanced metabolic efficiency with reduced resting energy expenditure due to weight loss and altered fuel utilization (favoring carbohydrate oxidation)^34^. This, combined with an increased drive to eat (hyperphagic response), promotes weight regain, particularly when the motivation for restricting caloric intake is lower^34^.

The mechanism behind the associations of increased body weight variability with cardiovascular events and mortality in NAFLD patients remains unclear. However, there are several plausible hypotheses. First, adipose tissue expands more rapidly with weight variability because of metabolic shifts favoring lipid storage^35^. Lipid accumulation induces excess hepatic lipid accumulation and often causes insulin resistance and chronic inflammation. In addition, animal and human studies have shown that weight fluctuations per se are related to an increased risk of developing hyperinsulinemia and insulin resistance^36,37^. Increased insulin resistance plays a crucial role in the progression of NAFLD^38,39^, which is related to adverse health outcomes. Second, weight fluctuations have been linked to several indicators of cardiometabolic disorders associated with an elevated risk of mortality. For example, weight fluctuations are associated with an increased C-reactive protein level^40^ and a lower high-density lipoprotein-cholesterol level^41^. Third, weight fluctuations may be related to a change in immune function^42^, as shown in a study reporting an association between repetitive episodic weight loss and reduced natural killer cell-mediated cytotoxicity^43^. Finally, weight variability can lead to sarcopenia via a loss of lean muscle mass and replacing fat mass for fat-free mass during weight regain. Sarcopenia is an independent risk factor for significant fibrosis in NAFLD^44^, and is also associated with CVD^45^.

We also investigated the impact of coexisting advanced liver fibrosis and the highest weight variability on the risk of CVD and mortality. The synergistic unfavorable influence of coexisting advanced liver fibrosis and the highest weight variability on CVD and mortality risk was identified in this study. Compared with controls without advanced liver fibrosis and the highest weight variability, individuals with both had an approximately 1.06-fold higher risk of MI, 1.34-fold higher risk of stroke, and 1.76-fold higher risk of all-cause mortality, even after adjusting for potential confounders. Advanced fibrosis is important risk factor of CVD and mortality in NAFLD patients^46,47^. Thus, our data suggest that it is particularly important that patients with NAFLD and significant liver fibrosis is especially needed to maintain normal body weight to prevent CVD and mortality.

Associations of weight variability with stroke and all-cause mortality were stronger in non-obese than obese NAFLD patients in the subgroup analyses. MI development was not associated with high weight variability in non-obese NAFLD patients. Bangalore et al.^16^ reported consistent findings of no association between high body weight variability and an increased risk of coronary events among normal-weight subjects. Although non-obese NAFLD patients tend to receive a better prognosis than obese NAFLD patients, they have a comparable CVD risk if they have advanced fibrosis^48^. This explains the associations of weight variability with CVD and mortality in non-obese and obese NAFLD patients. Nonsmokers may be more sensitive than former or current smokers to the effect of weight variability on the development of MI in NAFLD patients. Smoking is a major risk factor for CVD and mortality^49,50^. Our results indicate that high weight variability is a risk factor for CVD in NAFLD patients, even in nonsmokers, who normally are at lower risk of developing CVD. Further studies are warranted to confirm these findings.

The current study has several notable strengths. First, we demonstrated associations of body weight variability with CVD and mortality in a large sample size of > 720,000 individuals after a long follow-up of > 7 years, using a well-established and validated longitudinal national database. Second, we adjusted for potential confounding factors that potentially influence the associations between weight variability and long-term outcomes, including baseline BMI, to clarify the associations. In addition, various subgroup analyses were performed using nationwide cohort data, which supported the robustness of our main findings and provided interesting results. Third, because the NHIS cohort includes only Koreans, heterogeneity in the results induced by racial differences was avoided. Finally, our results suggest identifying a high-risk group in patients with metabolic dysfunction-associated fatty liver disease (MAFLD) using body weight variability. The term MAFLD was recently coined to reflect the undisputed role played by metabolic dysfunction in fatty liver disease^51,52^. Recent reports found that the MAFLD diagnosis criteria is more likely to capture high-risk groups with hepatic and extra-hepatic complications, supporting the change from NAFLD to MAFLD^53–55^. A significant positive association between body weight variability and the study outcome was consistent in subgroups with obesity, diabetes, hypertension, and dyslipidemia. Based on our results, the prognostic implication of body weight variability in patients with MAFLD is worthy of study.

Despite these strengths, our study also has some limitations. First, fatty liver was defined using the FLI in our population-based study. The FLI is a formula based on the BMI, waist circumference, triglyceride and gamma-glutamyl transferase levels, and the area under the receiver operator characteristic curve (AUROC) was 0.84 when the FLI was used to predict fatty liver in a cohort of 496 patients^56^. Subsequent validation studies revealed similar AUROCs of 0.81–0.89^57–60^, and the FLI was independently associated with outcomes related to NAFLD^61,62^. However, fatty liver as defined by the FLI could not avoid misclassification of the true presence of fatty liver. The FLI was developed using ultrasound as the reference and not the gold standard of liver biopsy. A FLI cut-off of 60 afforded a sensitivity of 60–70% when predicting fatty liver^57,60^, suggesting that a substantial number of NAFLD subjects might have been missed. Furthermore, the BMI and waist circumference cut-offs for obesity are lower in Asian populations compared to Western ones. Accordingly, recent studies have suggested that a lower FLI cut-off should be used when defining fatty liver in Asian populations^58,63^. Validation using liver imaging or histological data was lacking in our current study. Our results should be validated in patients with ultrasound or biopsy-proven NAFLD.

Second, we defined advanced fibrosis using the BARD score. That score showed an AUROC of 0.81 and negative predictive value of 96% for predicting advanced fibrosis in its initial report using a Western cohort^20^. Although the BARD score revealed similarly high AUROC and negative predictive values in another cohort of Caucasians^64^ and has been used to assess liver fibrosis^65,66^, we acknowledge that the use of other non-invasive markers including the fibrosis-4 index (FIB-4) and NAFLD fibrosis score (NFS) would have strengthened our results. Among non-invasive markers of fibrosis, the FIB-4 and NFS better assessed advanced fibrosis than did the BARD score^4,12,67–69^, and independently predicted CVD in patients with NAFLD^70^. Furthermore, the FIB-4 and NFS showed acceptable diagnostic performance when used to exclude advanced fibrosis regardless of elevated transaminase^71^ or diabetes^72^ status in patients with biopsy-proven NAFLD. However, we could not calculate the FIB-4 or NFS because we lacked data platelet counts and albumin levels.

Another limitation of the BARD score is that its clinical utility in Asian populations is debated. The BARD score is consisted of the BMI, aspartate aminotransferase/alanine aminotransferase ratio, and diabetes combined in a weighted sum. The BMI cut-off of 28 kg/m^2^ may be high for Asian populations, resulting in an underestimation of advanced fibrosis in Asians. Accordingly, external validation studies of the BARD score in Asian cohorts revealed lower AUROCs of 0.59–0.61^73,74^.

Non-invasive markers of fibrosis including the FIB-4, NFS, and BARD score yield high negative predictive values but low positive predictive values when employed to predict advanced liver fibrosis^20,67,75^. Thus, the main clinical utility of these markers is their ability to exclude subjects with advanced fibrosis, rather than to identify such subjects^75–77^. In addition, the predictive accuracy for advanced fibrosis was low in lean and morbidly obese patients^78^, and different according to the age^79^. Taken together, we might have missed some patients with advanced fibrosis. The association that we found between body weight variability and the risk outcomes according to advanced liver fibrosis should be validated in biopsy-proven NAFLD patients.

Third, because of the retrospective nature of this study, reverse causation may have been at play in our results. However, we considered the washout period when assessing study outcomes to address this issue. Our sensitivity analysis results with a 3-year lag time were consistent with our main findings. Fourth, because the study population was limited to Koreans, future studies in other ethnic groups are needed to generalize our results. In addition, approximately 85% of our NAFLD subjects were men; our results should be validated in women subjects with NAFLD to be applied to general populations.

In conclusion, in this nationwide, population-based study conducted in South Korea, body weight variability was independently associated with increased risks of MI, stroke, and all-cause mortality in patients with NAFLD. Overall, appropriate interventions for maintaining a normal body weight are needed to prevent future adverse health outcomes in NAFLD patients.

## Supplementary Information

Supplementary Tables.

Supplementary Figure 1.

## Abbreviations

AUROC: Area under the receiver operator characteristic curve
ARV: Average real variability
BMI: Body mass index
CVD: Cardiovascular disease
CV: Coefficient of variation
CI: Confidence interval
FIB-4: Fibrosis-4 index
NFS: NAFLD fibrosis score
FLI: Fatty liver index
HR: Hazard ratio
NHIC: National Health Insurance Corporation
NHIS: National Health Insurance Service
NAFLD: Nonalcoholic fatty liver disease
SD: Standard deviation
VIM: Variability independent of the mean
