# Supplementary material for: Body weight variability and the risk of cardiovascular outcomes in patients with nonalcoholic fatty liver disease
Source: Sci Rep. 2021 Apr 28;11:9154. doi: 10.1038/s41598-021-88733-3 (PMC8080815; doi:10.1038/s41598-021-88733-3)
Supplement: Supplementary file 1 — Supplementary Tables. [file 41598_2021_88733_MOESM1_ESM.docx]

**Body Weight Variability and the Risk of Cardiovascular Outcomes in Patients with Nonalcoholic Fatty Liver Disease**

Mi Na Kim*^1,2^, Kyungdo Han^3^, Juhwan Yoo^4^, Yeonjung Ha^1^, Young Eun Chon^1^, Ju Ho Lee^1^, Tracey G. Simon^5,6,7^, Andrew T. Chan^5,6^, and Seong Gyu Hwang*^1^

^1^Division of Gastroenterology, Department of Internal Medicine, CHA Bundang Medical Center, CHA University School of Medicine, Seongnam, Republic of Korea

^2^Clinical and Translational Hepatology Laboratory, Seongnam, Republic of Korea

^3^Department of Statistics and Actuarial Science, Soongsil University, Seoul, Republic of Korea

^4^Department of Biomedicine & Health Science, the Catholic University of Korea, Seoul, Korea

^5^Clinical and Translational Epidemiology Unit, Massachusetts General Hospital and Harvard Medical School, Boston, Massachusetts

^6^Division of Gastroenterology, Massachusetts General Hospital and Harvard Medical School, Boston, Massachusetts

^7^Liver Center, Division of Gastroenterology, Department of Medicine, Massachusetts General Hospital and Harvard Medical School, Boston, Massachusetts

Legend of Supplementary figure

Supplementary figure 1. Flow chart of study population

| Supplementary table 1. Prediction models for determining NAFLD or significant liver fibrosis | | | |
| --- | --- | --- | --- |
| Model |  | Cut-off point | Equation |
| Fatty liver index | determining NAFLD | ≥60 | (e^0.953×loge(triglycerides)+0.139×BMI+0.718×loge(GGT)+0.053×waistcircumference−15.745)^/ (1 +e^0.953×loge(triglycerides)+0.139×BMI+0.718×loge(GGT)+0.053×waistcircumference−15.745^) × 100 |
| BARD score | determining significant liver fibrosis | ≥2 | A weighted sum (AST/ALT ratio ≥0.8–2 points; a BMI ≥28 – 1 point; and the presence of diabetes – 1 point),  the possible score ranges from 0 to 4 points. |
| BMI, body mass index; ALT, alanine aminotransferase; AST, aspartate aminotransferase, GGT, gamma-glutamyl transpeptidase | | | |

| Supplementary table 2. Risks of outcomes with respect to quartiles of indices for body weight variability | | | | | | | | |
| --- | --- | --- | --- | --- | --- | --- | --- | --- |
|  |  | | | |  | HR (95% CI) | | |
|  | n | Event | Person-years | Incidence-rate^*^ |  | Model 1† | Model 2‡ | Model 3§ |
| **Standard deviation** |  |  |  |  |  |  |  |  |
| **Myocardial infarction** |  |  |  |  |  |  |  |  |
| Q1 | 192234 | 3276 | 1503335.07 | 2.17915 |  | 1(Ref.) | 1(Ref.) | 1(Ref.) |
| Q2 | 170140 | 2637 | 1351727.85 | 1.95084 |  | 1.035(0.984,1.09) | 1.025(0.974,1.079) | 1.025(0.974,1.079) |
| Q3 | 185999 | 3010 | 1463693.75 | 2.05644 |  | 1.115(1.061,1.171) | 1.098(1.044,1.153) | 1.097(1.044,1.153) |
| Q4 | 178363 | 2435 | 1400043.5 | 1.73923 |  | 1.138(1.079,1.2) | 1.102(1.046,1.162) | 1.102(1.045,1.163) |
| *P* for trend |  |  |  |  |  | <.0001 | <.0001 | <.0001 |
| **Stroke** |  |  |  |  |  |  |  |  |
| Q1 | 192234 | 4358 | 1499847.58 | 2.90563 |  | 1(Ref.) | 1(Ref.) | 1(Ref.) |
| Q2 | 170140 | 3333 | 1349090.02 | 2.47055 |  | 1.061(1.014,1.11) | 1.053(1.007,1.102) | 1.056(1.009,1.105) |
| Q3 | 185999 | 3838 | 1460718.74 | 2.62747 |  | 1.125(1.077,1.175) | 1.111(1.064,1.161) | 1.12(1.072,1.17) |
| Q4 | 178363 | 3185 | 1397617.56 | 2.27888 |  | 1.216(1.161,1.272) | 1.186(1.132,1.241) | 1.209(1.155,1.266) |
| *P* for trend |  |  |  |  |  | <.0001 | <.0001 | <.0001 |
| **All-cause mortality** |  |  |  |  |  |  |  |  |
| Q1 | 192234 | 6194 | 1513992.72 | 4.09117 |  | 1(Ref.) | 1(Ref.) | 1(Ref.) |
| Q2 | 170140 | 4738 | 1360232.81 | 3.48323 |  | 1.068(1.029,1.11) | 1.06(1.021,1.101) | 1.067(1.027,1.108) |
| Q3 | 185999 | 5713 | 1473292.91 | 3.87771 |  | 1.182(1.14,1.225) | 1.167(1.126,1.21) | 1.188(1.146,1.232) |
| Q4 | 178363 | 5519 | 1407617.22 | 3.92081 |  | 1.507(1.453,1.562) | 1.473(1.42,1.527) | 1.538(1.483,1.596) |
| *P* for trend |  |  |  |  |  | <.0001 | <.0001 | <.0001 |
| **Coefficient of variation** |  |  |  |  |  |  |  |  |
| **Myocardial infarction** |  |  |  |  |  |  |  |  |
| Q1 | 181635 | 2903 | 1424974.67 | 2.03723 |  | 1(Ref.) | 1(Ref.) | 1(Ref.) |
| Q2 | 181879 | 2845 | 1438517.13 | 1.97773 |  | 1.037(0.985,1.092) | 1.028(0.976,1.083) | 1.028(0.976,1.083) |
| Q3 | 181499 | 2898 | 1431759.86 | 2.02408 |  | 1.106(1.051,1.165) | 1.091(1.036,1.149) | 1.091(1.036,1.149) |
| Q4 | 181723 | 2712 | 1423548.5 | 1.9051 |  | 1.117(1.06,1.177) | 1.091(1.035,1.15) | 1.091(1.035,1.149) |
| *P* for trend |  |  |  |  |  | <.0001 | 0.0001 | 0.0001 |
| **Stroke** |  |  |  |  |  |  |  |  |
| Q1 | 181635 | 3721 | 1422355.83 | 2.61608 |  | 1(Ref.) | 1(Ref.) | 1(Ref.) |
| Q2 | 181879 | 3516 | 1436149 | 2.44821 |  | 1.025(0.979,1.074) | 1.018(0.973,1.066) | 1.017(0.971,1.065) |
| Q3 | 181499 | 3612 | 1429050.01 | 2.52755 |  | 1.103(1.053,1.154) | 1.09(1.041,1.141) | 1.089(1.04,1.14) |
| Q4 | 181723 | 3865 | 1419719.05 | 2.72237 |  | 1.241(1.186,1.298) | 1.221(1.167,1.277) | 1.222(1.168,1.278) |
| *P* for trend |  |  |  |  |  | <.0001 | <.0001 | <.0001 |
| **All-cause mortality** |  |  |  |  |  |  |  |  |
| Q1 | 181635 | 5132 | 1434609.56 | 3.57728 |  | 1(Ref.) | 1(Ref.) | 1(Ref.) |
| Q2 | 181879 | 5003 | 1447608.34 | 3.45605 |  | 1.071(1.03,1.113) | 1.063(1.023,1.106) | 1.059(1.019,1.101) |
| Q3 | 181499 | 5473 | 1440983.08 | 3.7981 |  | 1.231(1.185,1.279) | 1.214(1.168,1.261) | 1.209(1.164,1.256) |
| Q4 | 181723 | 6556 | 1431934.67 | 4.57842 |  | 1.558(1.502,1.616) | 1.528(1.473,1.585) | 1.524(1.469,1.581) |
| *P* for trend |  |  |  |  |  | <.0001 | <.0001 | <.0001 |
| **Average real variability** |  |  |  |  |  |  |  |  |
| **Myocardial infarction** |  |  |  |  |  |  |  |  |
| Q1 | 181796 | 2914 | 1435946.48 | 2.02932 |  | 1(Ref.) | 1(Ref.) | 1(Ref.) |
| Q2 | 204647 | 3276 | 1611608.75 | 2.03275 |  | 1.036(0.985,1.089) | 1.032(0.981,1.084) | 1.032(0.981,1.084) |
| Q3 | 163374 | 2518 | 1288763.96 | 1.95381 |  | 1.093(1.036,1.153) | 1.079(1.023,1.138) | 1.079(1.023,1.138) |
| Q4 | 176919 | 2650 | 1382480.98 | 1.91684 |  | 1.166(1.106,1.229) | 1.133(1.075,1.194) | 1.133(1.074,1.195) |
| *P* for trend |  |  |  |  |  | <.0001 | <.0001 | <.0001 |
| **Stroke** |  |  |  |  |  |  |  |  |
| Q1 | 181796 | 3769 | 1433135.38 | 2.6299 |  | 1(Ref.) | 1(Ref.) | 1(Ref.) |
| Q2 | 204647 | 4211 | 1608326.06 | 2.61825 |  | 1.026(0.982,1.072) | 1.023(0.979,1.069) | 1.026(0.982,1.072) |
| Q3 | 163374 | 3273 | 1285991.17 | 2.54512 |  | 1.123(1.072,1.177) | 1.112(1.061,1.166) | 1.122(1.07,1.176) |
| Q4 | 176919 | 3461 | 1379821.28 | 2.5083 |  | 1.19(1.136,1.246) | 1.162(1.11,1.217) | 1.183(1.129,1.239) |
| *P* for trend |  |  |  |  |  | <.0001 | <.0001 | <.0001 |
| **All-cause mortality** |  |  |  |  |  |  |  |  |
| Q1 | 181796 | 5238 | 1445474.55 | 3.62372 |  | 1(Ref.) | 1(Ref.) | 1(Ref.) |
| Q2 | 204647 | 6134 | 1622251.91 | 3.78116 |  | 1.079(1.04,1.119) | 1.075(1.036,1.115) | 1.083(1.044,1.124) |
| Q3 | 163374 | 4892 | 1296668.75 | 3.77274 |  | 1.207(1.161,1.255) | 1.197(1.151,1.244) | 1.22(1.174,1.269) |
| Q4 | 176919 | 5900 | 1390740.45 | 4.24234 |  | 1.465(1.412,1.521) | 1.434(1.381,1.488) | 1.491(1.436,1.548) |
| *P* for trend |  |  |  |  |  | <.0001 | <.0001 | <.0001 |
| *Incidence per 1,000 person-years. | |  |  |  |  |  |  |  |
| †Model 1 was adjusted for age and sex. | |  |  |  |  |  |  |  |
| ‡Model 2 was further adjusted for age, sex, smoking status, alcohol consumption, physical activity, hypertension, diabetes, dyslipidemia, chronic kidney disease. | | | | | | | | |
| §Model 3 was further adjusted for age, sex, smoking status, alcohol consumption, physical activity, hypertension, diabetes, dyslipidemia, chronic kidney disease, and baseline BMI. | | | | | | | | |

| \| Supplementary table 3. Continuous body weight variability and risks of outcomes \| \| \| \| \|  \|  \|  \| \| --- \| --- \| --- \| --- \| --- \| --- \| --- \| --- \| \|  \|  \| HR (95% CI) \| \| \| \| \| \| \|  \|  \| Model 1† \| *P* value \| Model 2‡ \| *P* value \| Model 3§ \| *P* value \| \| **Myocardial infarction** \|  \|  \|  \|  \|  \|  \|  \| \| VIM \|  \| 1.024 (1.012,1.037) \| <.0001 \| 1.02 (1.007,1.032) \| 0.0016 \| 1.019 (1.007,1.032) \| 0.0019 \| \| Standard deviation \|  \| 1.021 (1.011,1.032) \| <.0001 \| 1.015 (1.005,1.027) \| 0.0054 \| 1.015 (1.004,1.027) \| 0.0071 \| \| Coefficient of variation \|  \| 1.016 (1.008,1.024) \| <.0001 \| 1.013 (1.005,1.021) \| 0.0012 \| 1.013 (1.005,1.021) \| 0.0014 \| \| Average real variability \|  \| 1.022(1.013,1.031) \| <.0001 \| 1.017 (1.008,1.026) \| 0.0001 \| 1.017 (1.008,1.026) \| 0.0002 \| \| **Stroke** \|  \|  \|  \|  \|  \|  \|  \| \| VIM \|  \| 1.043 (1.033,1.052) \| <.0001 \| 1.04 (1.03,1.05) \| <.0001 \| 1.043 (1.033,1.054) \| <.0001 \| \| Standard deviation \|  \| 1.036 (1.027,1.046) \| <.0001 \| 1.032 (1.023,1.042) \| <.0001 \| 1.038 (1.029,1.048) \| <.0001 \| \| Coefficient of variation \|  \| 1.028 (1.022,1.034) \| <.0001 \| 1.027 (1.02,1.033) \| <.0001 \| 1.028 (1.022,1.035) \| <.0001 \| \| Average real variability \|  \| 1.03 (1.023,1.037) \| <.0001 \| 1.026 (1.019,1.034) \| <.0001 \| 1.03 (1.023,1.037) \| <.0001 \| \| **All-cause mortality** \|  \|  \|  \|  \|  \|  \|  \| \| VIM \|  \| 1.086 (1.08,1.093) \| <.0001 \| 1.084 (1.077,1.091) \| <.0001 \| 1.093 (1.085,1.1) \| <.0001 \| \| Standard deviation \|  \| 1.076 (1.07,1.083) \| <.0001 \| 1.073 (1.066,1.079) \| <.0001 \| 1.087 (1.08,1.094) \| <.0001 \| \| Coefficient of variation \|  \| 1.056 (1.051,1.06) \| <.0001 \| 1.054 (1.049,1.058) \| <.0001 \| 1.059 (1.054,1.063) \| <.0001 \| \| Average real variability \|  \| 1.057 (1.051,1.062) \| <.0001 \| 1.053 (1.048,1.058) \| <.0001 \| 1.061 (1.056,1.066) \| <.0001 \| \| †Model 1 was adjusted for age and sex. \| \| \|  \|  \|  \|  \|  \| \| ‡Model 2 was further adjusted for age, sex, smoking status, alcohol consumption, physical activity, hypertension, diabetes, dyslipidemia, and chronic kidney disease. \| \| \| \| \| \| \| \| \| §Model 3 was further adjusted for age, sex, smoking status, alcohol consumption, physical activity, hypertension, diabetes, dyslipidemia, chronic kidney disease, and baseline BMI. \| \| \| \| \| \| \| \|  \| Supplementary table 4. Risks of outcomes with respect to body weight variability (Q4 vs. Q1-3 of VIM) in subgroups \| \| \| \| \| \| \| \| \| \| \| \| \| \| --- \| --- \| --- \| --- \| --- \| --- \| --- \| --- \| --- \| --- \| --- \| --- \| --- \| \| Subgroup \| n (Q4) \| Event (Q4) \| MI \| \|  \| Event (Q4) \| Stroke \| \|  \| Event (Q4) \| All-cause mortality \| \| \| HR (95% CI)^*^ \| *P*  for interaction \|  \| HR (95% CI)^*^ \| *P*  for interaction \|  \| HR (95% CI)^*^ \| *P*  for interaction \| \| **Age** \|  \|  \|  \| 0.0007 \|  \|  \|  \| 0.0691 \|  \|  \|  \| 0.0368 \| \| 20-39 years \| 80897 \| 367 \| 0.949(0.837,1.077) \|  \|  \| 238 \| 0.941(0.804,1.102) \|  \|  \| 449 \| 1.202(1.067,1.355) \|  \| \| 40-64 years \| 82453 \| 1351 \| 1.031(0.971,1.095) \|  \|  \| 1700 \| 1.192(1.129,1.258) \|  \|  \| 2473 \| 1.451(1.386,1.518) \|  \| \| ≥65 years \| 18327 \| 950 \| 1.17(1.085,1.261) \|  \|  \| 1844 \| 1.233(1.167,1.302) \|  \|  \| 3448 \| 1.325(1.273,1.38) \|  \| \| **Sex** \|  \|  \|  \| 0.05 \|  \|  \|  \| 0.3873 \|  \|  \|  \| 0.4584 \| \| Men \| 147713 \| 1936 \| 1.02(0.97,1.073) \|  \|  \| 2579 \| 1.175(1.124,1.228) \|  \|  \| 4871 \| 1.408(1.362,1.455) \|  \| \| Women \| 33964 \| 732 \| 1.138(1.043,1.242) \|  \|  \| 1203 \| 1.214(1.134,1.301) \|  \|  \| 1499 \| 1.35(1.268,1.438) \|  \| \| **Smoking status** \|  \|  \|  \| 0.0095 \|  \|  \|  \| 0.842 \|  \|  \|  \| 0.4745 \| \| Nonsmoker \| 70351 \| 1234 \| 1.117(1.047,1.193) \|  \|  \| 1988 \| 1.18(1.12,1.242) \|  \|  \| 3095 \| 1.411(1.352,1.473) \|  \| \| Former smoker \| 38241 \| 523 \| 1.087(0.986,1.199) \|  \|  \| 738 \| 1.206(1.11,1.31) \|  \|  \| 1359 \| 1.403(1.318,1.493) \|  \| \| Current smoker \| 73085 \| 911 \| 0.945(0.878,1.017) \|  \|  \| 1056 \| 1.189(1.109,1.274) \|  \|  \| 1916 \| 1.366(1.296,1.439) \|  \| \| **Diabetes** \|  \|  \|  \| 0.3263 \|  \|  \|  \| 0.8508 \|  \|  \|  \| 0.6419 \| \| Yes \| 28465 \| 815 \| 1.089(1.006,1.18) \|  \|  \| 1368 \| 1.205(1.132,1.283) \|  \|  \| 2263 \| 1.412(1.343,1.483) \|  \| \| No \| 153212 \| 1853 \| 1.038(0.985,1.093) \|  \|  \| 2414 \| 1.181(1.128,1.237) \|  \|  \| 4107 \| 1.392(1.343,1.443) \|  \| \| **Hypertension** \|  \|  \|  \| 0.1311 \|  \|  \|  \| 0.417 \|  \|  \|  \| 0.647 \| \| Yes \| 72632 \| 1675 \| 1.082(1.024,1.143) \|  \|  \| 2688 \| 1.207(1.155,1.261) \|  \|  \| 4302 \| 1.394(1.345,1.445) \|  \| \| No \| 109045 \| 993 \| 1.013(0.943,1.088) \|  \|  \| 1094 \| 1.163(1.086,1.245) \|  \|  \| 2068 \| 1.414(1.344,1.488) \|  \| \| **Dyslipidemia** \|  \|  \|  \| 0.5403 \|  \|  \|  \| 0.7783 \|  \|  \|  \| 0.1431 \| \| Yes \| 133836 \| 1576 \| 1.037(0.98,1.098) \|  \|  \| 1469 \| 1.198(1.128,1.271) \|  \|  \| 2122 \| 1.356(1.289,1.426) \|  \| \| No \| 47841 \| 1092 \| 1.072(1.002,1.148) \|  \|  \| 2313 \| 1.183(1.128,1.24) \|  \|  \| 4248 \| 1.42(1.37,1.472) \|  \| \| **Alcohol consumption** \|  \|  \|  \| 0.1334 \|  \|  \|  \| 0.8049 \|  \|  \|  \| 0.8861 \| \| none \| 69785 \| 1496 \| 1.087(1.024,1.153) \|  \|  \| 2154 \| 1.197(1.139,1.259) \|  \|  \| 3474 \| 1.393(1.338,1.45) \|  \| \| ≤210 g/week (30 g/day) for men,   ≤140 g/week (20 g/day) for women \| 111892 \| 1172 \| 1.005(0.942,1.072) \|  \|  \| 1628 \| 1.178(1.115,1.245) \|  \|  \| 2896 \| 1.397(1.339,1.458) \|  \| \| **BMI** \|  \|  \|  \| 0.233 \|  \|  \|  \| 0.0283 \|  \|  \|  \| 0.0184 \| \| BMI <25 kg/m^2^ \| 21790 \| 374 \| 0.949(0.845,1.066) \|  \|  \| 715 \| 1.246(1.141,1.361) \|  \|  \| 1579 \| 1.357(1.276,1.443) \|  \| \| BMI ≥25 kg/m2 \| 159887 \| 2294 \| 1.059(1.01,1.11) \|  \|  \| 3067 \| 1.159(1.112,1.208) \|  \|  \| 4791 \| 1.346(1.301,1.392) \|  \| \| *Adjusted for age, sex, smoking status, alcohol consumption, physical activity, hypertension, diabetes, dyslipidemia, chronic kidney disease, and baseline BMI. \| \| \| \| \| \| \| \| \| \| \|  \|  \|   Supplementary table 5. Risks of outcomes with 3-year of lag time with respect to quartiles of VIM for body weight | | | | | | | | | | | | | | | | |
| --- | --- | --- | --- | --- | --- | --- | --- | --- | --- | --- | --- | --- | --- | --- | --- | --- | --- | --- | --- | --- | --- | --- | --- | --- | --- | --- | --- | --- | --- | --- | --- | --- | --- | --- | --- | --- | --- | --- | --- | --- | --- | --- | --- | --- | --- | --- | --- | --- | --- | --- | --- | --- | --- | --- | --- | --- | --- | --- | --- | --- | --- | --- | --- | --- | --- | --- | --- | --- | --- | --- | --- | --- | --- | --- | --- | --- | --- | --- | --- | --- | --- | --- | --- | --- | --- | --- | --- | --- | --- | --- | --- | --- | --- | --- | --- | --- | --- | --- | --- | --- | --- | --- | --- | --- | --- | --- | --- | --- | --- | --- | --- | --- | --- | --- | --- | --- | --- | --- | --- | --- | --- | --- | --- | --- | --- | --- | --- | --- | --- | --- | --- | --- | --- | --- | --- | --- | --- | --- | --- | --- | --- | --- | --- | --- | --- | --- | --- | --- | --- | --- | --- | --- | --- | --- | --- | --- | --- | --- | --- | --- | --- | --- | --- | --- | --- | --- | --- | --- | --- | --- | --- | --- | --- | --- | --- | --- | --- | --- | --- | --- | --- | --- | --- | --- | --- | --- | --- | --- | --- | --- | --- | --- | --- | --- | --- | --- | --- | --- | --- | --- | --- | --- | --- | --- | --- | --- | --- | --- | --- | --- | --- | --- | --- | --- | --- | --- | --- | --- | --- | --- | --- | --- | --- | --- | --- | --- | --- | --- | --- | --- | --- | --- | --- | --- | --- | --- | --- | --- | --- | --- | --- | --- | --- | --- | --- | --- | --- | --- | --- | --- | --- | --- | --- | --- | --- | --- | --- | --- | --- | --- | --- | --- | --- | --- | --- | --- | --- | --- | --- | --- | --- | --- | --- | --- | --- | --- | --- | --- | --- | --- | --- | --- | --- | --- | --- | --- | --- | --- | --- | --- | --- | --- | --- | --- | --- | --- | --- | --- | --- | --- | --- | --- | --- | --- | --- | --- | --- | --- | --- | --- | --- | --- | --- | --- | --- | --- | --- | --- | --- | --- | --- | --- | --- | --- | --- | --- | --- | --- | --- | --- | --- | --- | --- | --- | --- | --- | --- | --- | --- | --- | --- | --- | --- | --- | --- | --- | --- | --- | --- | --- | --- | --- | --- | --- | --- | --- | --- | --- | --- | --- | --- | --- | --- | --- | --- | --- | --- | --- | --- | --- | --- | --- | --- | --- | --- | --- | --- | --- | --- | --- | --- | --- | --- | --- | --- | --- | --- | --- | --- | --- | --- | --- | --- | --- | --- | --- | --- | --- | --- | --- | --- | --- | --- | --- | --- | --- | --- | --- | --- | --- | --- | --- | --- | --- | --- | --- | --- | --- | --- | --- | --- | --- | --- | --- | --- | --- | --- | --- | --- | --- | --- | --- | --- | --- | --- | --- | --- | --- | --- | --- | --- | --- | --- | --- | --- | --- | --- | --- | --- | --- | --- | --- | --- | --- | --- | --- | --- | --- | --- | --- | --- | --- | --- | --- | --- | --- | --- | --- | --- | --- | --- | --- | --- | --- | --- | --- | --- | --- | --- | --- | --- | --- | --- | --- | --- | --- | --- | --- | --- | --- | --- | --- | --- | --- | --- | --- | --- | --- | --- | --- | --- | --- | --- | --- | --- | --- | --- | --- | --- | --- | --- | --- | --- | --- | --- | --- | --- | --- | --- | --- | --- | --- | --- | --- | --- | --- | --- | --- | --- | --- | --- | --- | --- | --- | --- | --- | --- | --- | --- | --- | --- | --- | --- | --- | --- | --- | --- | --- | --- | --- | --- | --- | --- | --- | --- | --- | --- | --- | --- | --- | --- | --- | --- | --- | --- | --- | --- | --- | --- |
|  |  | | | | | | | |  | HR (95% CI) | | | | | | |
|  | n | Event | | Person-years | | | Incidence-rate^*^ | |  | Model 1† | | | Model 2‡ | | Model 3§ | |
| **Myocardial infarction** |  |  | |  | | |  | |  |  | | |  | |  | |
| Q1 | 178702 | 1990 | | 878310.05 | | | 2.26571 | |  | 1(Ref.) | | | 1(Ref.) | | 1(Ref.) | |
| Q2 | 178454 | 1950 | | 889008.51 | | | 2.19345 | |  | 1.041(0.978,1.108) | | | 1.032(0.97,1.099) | | 1.033(0.97,1.099) | |
| Q3 | 178475 | 1997 | | 885873.07 | | | 2.25427 | |  | 1.128(1.06,1.2) | | | 1.113(1.046,1.185) | | 1.113(1.046,1.184) | |
| Q4 | 178286 | 1822 | | 877044.21 | | | 2.07743 | |  | 1.136(1.066,1.211) | | | 1.111(1.043,1.184) | | 1.109(1.04,1.182) | |
| *P* for trend |  |  | |  | | |  | |  | <.0001 | | | 0.0001 | | 0.0002 | |
| **Stroke** |  |  | |  | | |  | |  |  | | |  | |  | |
| Q1 | 178702 | 2551 | | 877001.13 | | | 2.90878 | |  | 1(Ref.) | | | 1(Ref.) | | 1(Ref.) | |
| Q2 | 178454 | 2352 | | 888027.58 | | | 2.64857 | |  | 1.008(0.953,1.066) | | | 1.002(0.948,1.06) | | 1.001(0.947,1.059) | |
| Q3 | 178475 | 2356 | | 884888.88 | | | 2.66248 | |  | 1.075(1.016,1.137) | | | 1.064(1.006,1.125) | | 1.065(1.007,1.126) | |
| Q4 | 178286 | 2504 | | 875432.32 | | | 2.8603 | |  | 1.244(1.177,1.315) | | | 1.225(1.159,1.295) | | 1.23(1.164,1.3) | |
| *P* for trend |  |  | |  | | |  | |  | <.0001 | | | <.0001 | | <.0001 | |
| **All-cause mortality** |  |  | |  | | |  | |  |  | | |  | |  | |
| Q1 | 178702 | 3809 | | 882398.04 | | | 4.31665 | |  | 1(Ref.) | | | 1(Ref.) | | 1(Ref.) | |
| Q2 | 178454 | 3657 | | 893035.34 | | | 4.09502 | |  | 1.067(1.02,1.117) | | | 1.06(1.013,1.109) | | 1.056(1.009,1.105) | |
| Q3 | 178475 | 3885 | | 889932 | | | 4.3655 | |  | 1.21(1.157,1.265) | | | 1.195(1.142,1.249) | | 1.195(1.143,1.25) | |
| Q4 | 178286 | 4446 | | 880660.09 | | | 5.04849 | |  | 1.505(1.442,1.572) | | | 1.479(1.416,1.544) | | 1.485(1.422,1.551) | |
| *P* for trend |  |  | |  | | |  | |  | <.0001 | | | <.0001 | | <.0001 | |
| *Incidence per 1,000 person-years. | | |  | |  |  | |  | | |  |  | |  | |  |
| †Model 1 was adjusted for age and sex. | | | | |  |  | |  | | |  |  | |  | |  |
| ‡Model 2 was further adjusted for age, sex, smoking status, alcohol consumption, physical activity, hypertension, diabetes, dyslipidemia, chronic kidney disease. | | | | | | | | | | | | | | | | |
| §Model 3 was further adjusted for age, sex, smoking status, alcohol consumption, physical activity, hypertension, diabetes, dyslipidemia, chronic kidney disease, and baseline BMI. | | | | | | | | | | | | | | | | |

| Supplementary table 6. Risks of outcomes with excluding cancer patients with respect to quartiles of VIM for body weight | | | | | | | | | | |
| --- | --- | --- | --- | --- | --- | --- | --- | --- | --- | --- |
|  |  | | | |  | HR (95% CI) | | | | |
|  | n | Event | Person-years | Incidence-rate^*^ |  | Model 1^†^ | Model 2^‡^ | | Model 3^§^ | |
| **Myocardial infarction** |  |  |  |  |  |  |  | |  | |
| Q1 | 180063 | 2921 | 1412499.95 | 2.06796 |  | 1(Ref.) | 1(Ref.) | | 1(Ref.) | |
| Q2 | 179761 | 2793 | 1422727.73 | 1.96313 |  | 1.022(0.97,1.076) | 1.012(0.961,1.066) | | 1.013(0.961,1.066) | |
| Q3 | 179886 | 2863 | 1419669.29 | 2.01667 |  | 1.106(1.05,1.164) | 1.09(1.035,1.147) | | 1.09(1.035,1.147) | |
| Q4 | 179560 | 2606 | 1408387.85 | 1.85034 |  | 1.107(1.05,1.167) | 1.079(1.024,1.138) | | 1.079(1.023,1.138) | |
| *P* for trend |  |  |  |  |  | <.0001 | 0.0003 | | 0.0004 | |
| **Stroke** |  |  |  |  |  |  |  | |  | |
| Q1 | 180063 | 3731 | 1409920.15 | 2.64625 |  | 1(Ref.) | 1(Ref.) | | 1(Ref.) | |
| Q2 | 179761 | 3482 | 1420289.98 | 2.45161 |  | 1.026(0.979,1.074) | 1.018(0.972,1.066) | | 1.017(0.971,1.065) | |
| Q3 | 179886 | 3498 | 1417230.23 | 2.46819 |  | 1.093(1.044,1.145) | 1.08(1.031,1.131) | | 1.081(1.032,1.132) | |
| Q4 | 179560 | 3682 | 1404770.89 | 2.62107 |  | 1.242(1.186,1.3) | 1.22(1.165,1.277) | | 1.224(1.17,1.282) | |
| *P* for trend |  |  |  |  |  | <.0001 | <.0001 | | <.0001 | |
| **All-cause mortality** |  |  |  |  |  |  |  | |  | |
| Q1 | 180063 | 5061 | 1422192.55 | 3.55859 |  | 1(Ref.) | 1(Ref.) | | 1(Ref.) | |
| Q2 | 179761 | 4919 | 1431719.16 | 3.43573 |  | 1.082(1.041,1.126) | 1.073(1.032,1.116) | | 1.069(1.028,1.112) | |
| Q3 | 179886 | 5259 | 1428757.8 | 3.68082 |  | 1.23(1.184,1.279) | 1.213(1.168,1.261) | | 1.214(1.168,1.261) | |
| Q4 | 179560 | 6044 | 1416476.19 | 4.26693 |  | 1.536(1.479,1.594) | 1.506(1.45,1.563) | | 1.511(1.456,1.569) | |
| *P* for trend |  |  |  |  |  | <.0001 | <.0001 | | <.0001 | |
| *Incidence per 1,000 person-years. |  |  |  |  |  |  | |  | |  |
| †Model 1 was adjusted for age and sex. | |  |  |  |  |  | |  | |  |
| ‡Model 2 was further adjusted for age, sex, smoking status, alcohol consumption, physical activity, hypertension, diabetes, dyslipidemia, chronic kidney disease. | | | | | | | | | | |

§Model 3 was further adjusted for age, sex, smoking status, alcohol consumption, physical activity, hypertension, diabetes, dyslipidemia, chronic kidney disease, and baseline BMI.
