# Supplementary figures and images for: Body weight variability and the risk of cardiovascular outcomes in patients with nonalcoholic fatty liver disease
Source: Sci Rep. 2021 Apr 28;11:9154. doi: 10.1038/s41598-021-88733-3 (PMC8080815; doi:10.1038/s41598-021-88733-3)

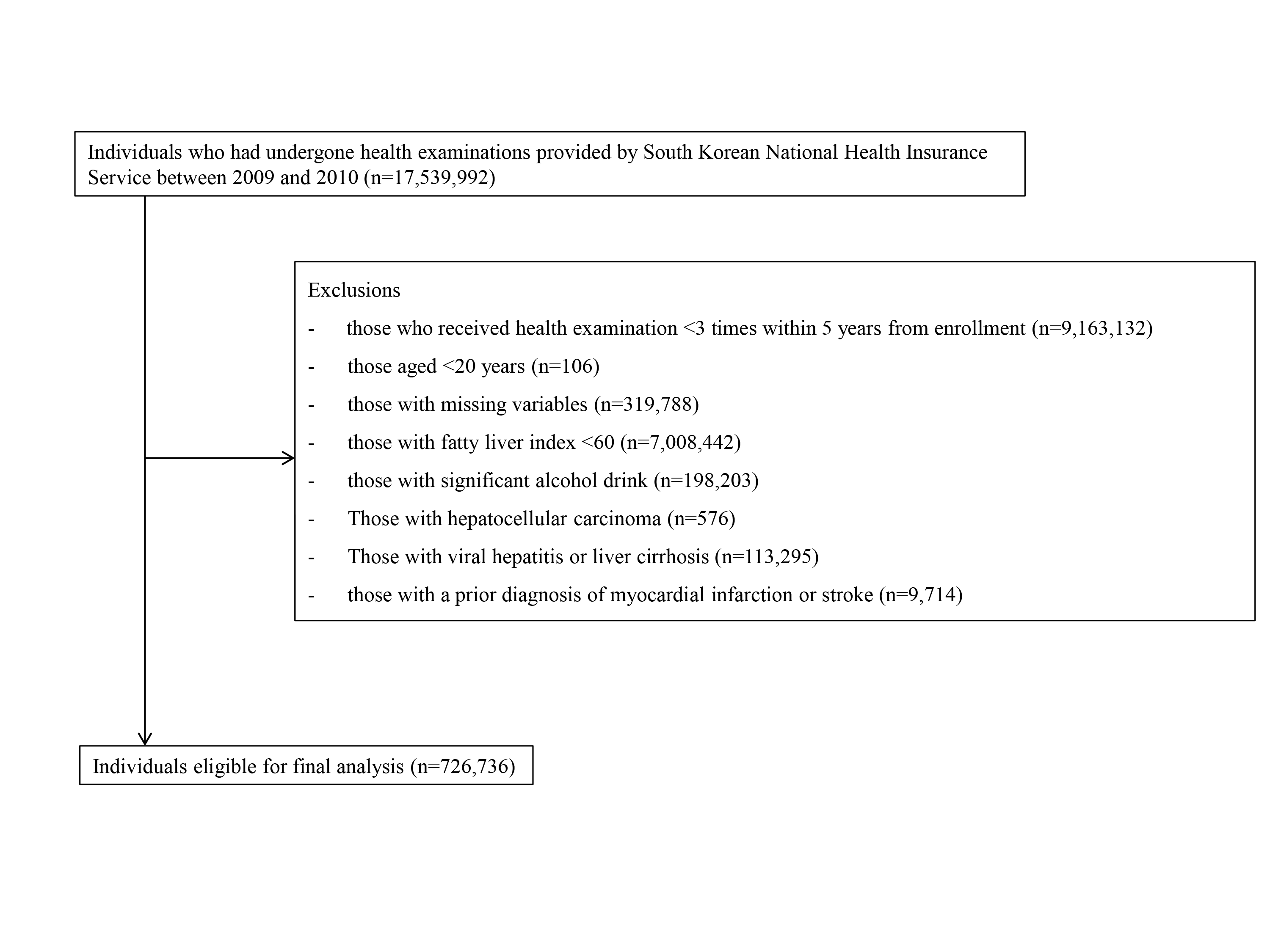

Supplement: Supplementary file 2 — Supplementary Figure 1. [file 41598_2021_88733_MOESM2_ESM.tif]
